# Severe Retinal Vascular Dysplasia in a Low-Birth-Weight Infant Born at 32 Weeks of Gestation: A Case Report

**DOI:** 10.7759/cureus.85431

**Published:** 2025-06-05

**Authors:** Mizuki Asano, Nozomi Matsumura, Kizuku Kumagai, Tomoko Ohno, Nobuhisa Mizuki

**Affiliations:** 1 Department of Ophthalmology, Kanagawa Children’s Medical Center, Yokohama, JPN; 2 Department of Ophthalmology and Visual Science, Yokohama City University School of Medicine, Yokohama, JPN

**Keywords:** aggressive posterior retinopathy of prematurity, arop, fluorescein angiography, optical coherence tomography, retinopathy of prematurity

## Abstract

Severe retinal vascular dysplasia resembling aggressive posterior retinopathy of prematurity (ROP) can occur even in moderately preterm infants. We report the case of a male infant born at 32 weeks and 1 day of gestation, weighing 861 g, who developed advanced retinal vascular malformations. Initial management included intubation for respiratory distress syndrome, but the patient stabilized without further complications. At 36 weeks and 5 days post-menstrual age, ophthalmological evaluation revealed vascular tortuosity, abnormal anastomoses, and extensive peripheral nonperfusion in both eyes. Fluorescein angiography (FA) identified avascular regions, guiding effective bilateral retinal photocoagulation with 885 and 1,776 laser spots applied to the right and left eyes, respectively. Post-treatment follow-up showed no recurrence of retinopathy, although macular changes, specifically temporal dragging and macular scarring, persisted in the left eye. Notably, this infant did not exhibit common ROP risk factors such as oxygen toxicity or cerebral hemorrhage. In addition, genetic and neurological evaluations were unremarkable. These findings suggest a distinct etiology, potentially related to retinal vascular dysplasia rather than classical ROP. This case highlights the diagnostic value of FA in identifying avascular retinal zones and guiding treatment, especially in atypical presentations. While optical coherence tomography angiography provides high-resolution vascular imaging, FA remains a practical and effective tool in pediatric settings. Even in moderately preterm infants, comprehensive ophthalmologic evaluation should be considered based on ocular findings and clinical context, and further research is warranted to elucidate the mechanisms underlying atypical retinal vascular dysplasias.

## Introduction

It is widely recognized that the risk of developing advanced forms of retinopathy of prematurity (ROP), including aggressive posterior ROP (AROP), is high in very preterm infants and those with very low birth weight [1,2]. The etiology of this condition is attributed to the presence of premature retinas in preterm infants who do not develop in a normal manner [1]. Specifically, underdeveloped blood vessels may exhibit aberrant growth patterns, resulting in retinal detachment from their typical anatomical position [3]. In full-term infants, retinal vascularization is complete and does not occur. The two most prevalent categories of ROP are the fulminant form (AROP) and the normal form (classic ROP) [4,5]. These two types of ROP exhibit divergent characteristics in terms of disease progression and treatment response [4]. Specifically, the classic ROP progresses gradually through five stages [6]. In contrast, fulminant ROP progresses rapidly over a brief period, often leading to rapid retinal detachment [7]. A comprehensive review of extant studies has identified extreme prematurity and low birth weight, oxygen administration, infection, and cerebral hemorrhage as risk factors for retinopathy of prematurity [8].

The primary treatments for ROP are laser photocoagulation and antivascular endothelial growth factor (VEGF) therapy [9]. VEGF is overexpressed in avascular areas of the immature retina, and laser photocoagulation targets these areas to suppress VEGF production [10,11]. Anti-VEGF agents, conversely, directly inhibit VEGF activity [12]. Accurate identification of avascular zones is essential for effective photocoagulation, as missed areas can continue to drive neovascularization [13].

This case study underscores the fact that, in addition to the observation that advanced retinal vascular dysplasia, such as AROP, can appear in children who are not born at the extremely premature stage of development, the utilization of fluorescein angiography (FA) in conjunction with a fundus examination enables a more accurate identification of avascular regions within the retina.

## Case presentation

We present a case of severe retinal vascular malformation resembling AROP in a male infant born at 32 weeks and 1 day of gestation with a birth weight of 861 g. He was delivered via emergency cesarean section due to non-reassuring fetal status. The neonate was admitted to the neonatal intensive care unit at Kanagawa Prefectural Children's Medical Center, where comprehensive systemic management was initiated.

The infant required intubation due to respiratory distress syndrome immediately after birth, but was successfully extubated on day one and remained stable in terms of respiratory function thereafter. Echocardiography revealed mesocardia and a left superior vena cava remnant; however, no therapeutic intervention was deemed necessary. Other findings included a suburethral fissure and an undescended testicle.

**Figure 1:**
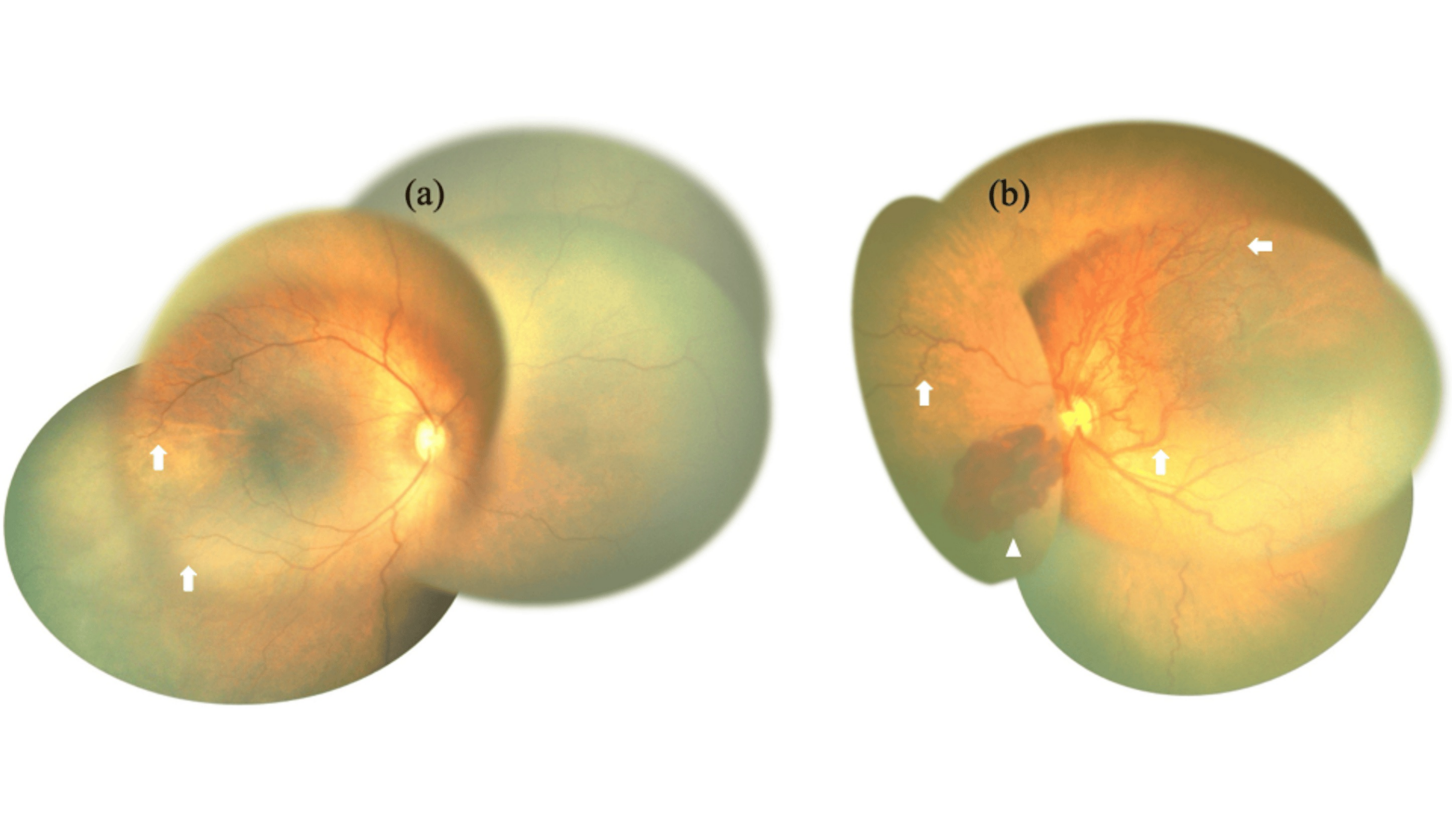
Fundus photograph at corrected 36 weeks and 5 days. (a) Disruption of the retinal vessels (arrows) on the auricular side can be observed in the photograph of the right eye. (b) A photograph of the left eye, showing dilatation and tortuosity of retinal vessels, an anastomosis of retinal vessels (arrows), and retinal hemorrhage (arrowheads) on the left nasal side.

**Figure 2:**
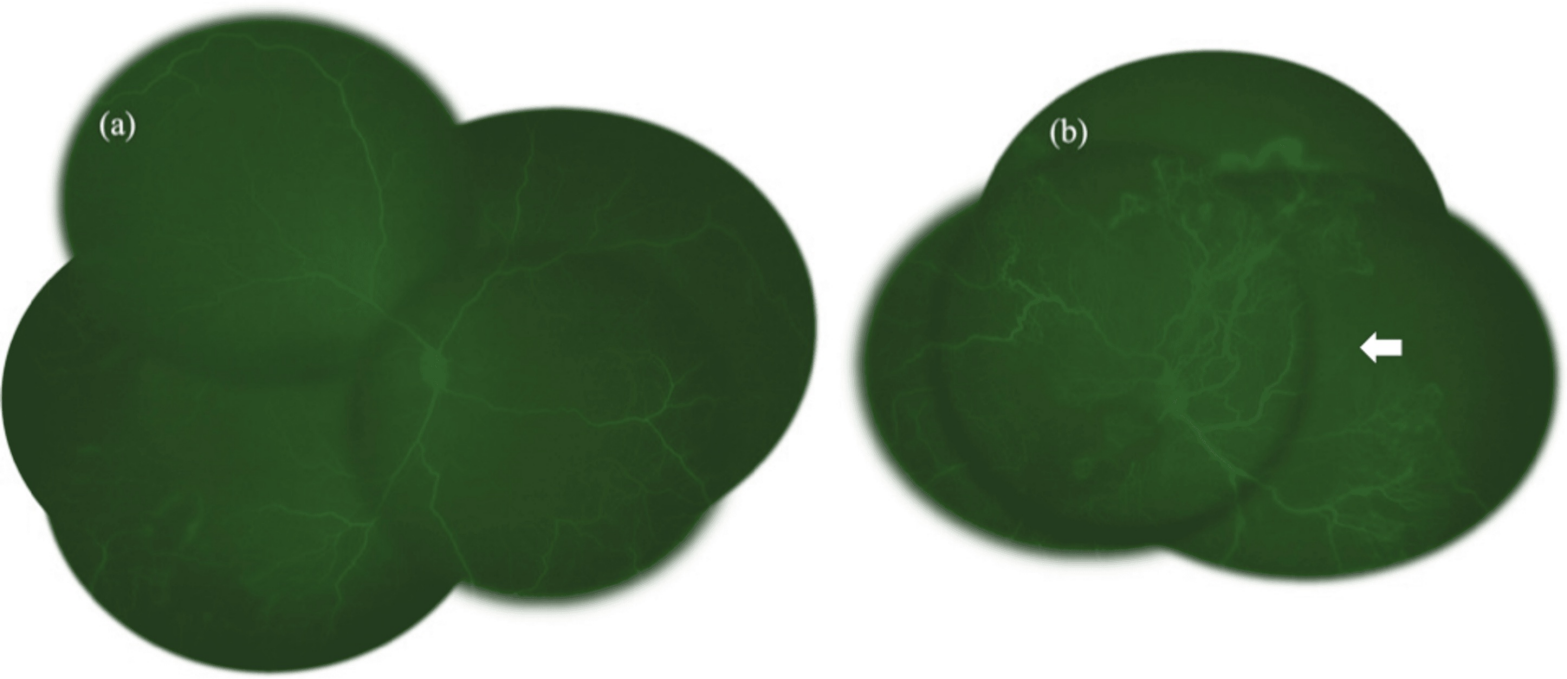
Fluorescence fundus angiography images. (a and b) Fluorescence fundus angiography images. A wide area of no perfusion is seen extending to the posterior pole, and in the left eye, the blood vessels do not extend even to the vicinity of the macula (arrows).

At 36 weeks and 5 days post-menstrual age, a detailed ophthalmological evaluation was performed due to the high risk of retinopathy in preterm infants. Fundoscopic examination revealed a cessation of retinal vascular growth in the temporal side of the right eye and vascular tortuosity and anomalous vascular anastomoses in the left eye, with bleeding on the nasal side (Figure 1). FA revealed peripheral retinal nonperfusion in the right eye. In the left eye, nonperfusion was observed over a wide area, extending from at least 9 o’clock to 4 o’clock, accompanied by abnormal vascular anastomoses. Notably, the temporal retinal vessels in the left eye extended only up to the zone I region (Figure 2).

**Figure 3:**
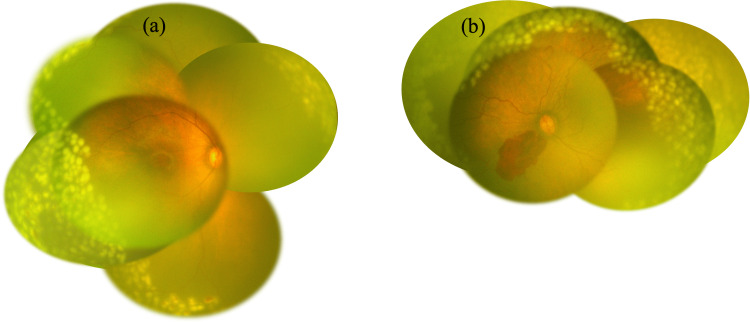
Images after retinal photocoagulation. (a and b) Funduscopic images taken immediately after retinal photocoagulation. Retinal photocoagulation was performed over the extensive retinal avascularity in both eyes (right eye: 885 spots, left eye: 1,776 spots).

**Figure 4:**
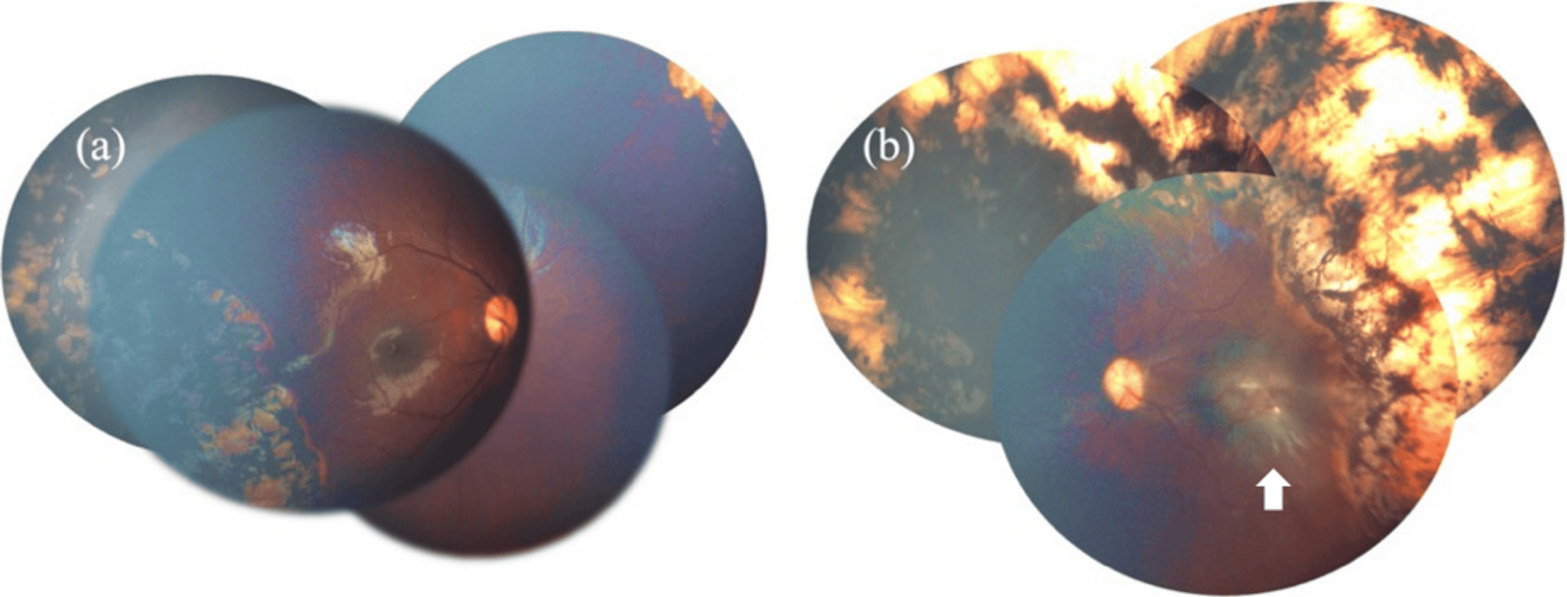
Fundus photograph two years after surgery. Postoperatively, retinal vasodilation has improved and there is no progression of retinopathy, although there are organic changes in the left macula (arrows). The right eye has good pursuit vision, and the left eye has no pursuit vision but retains light perception.

Given these findings, extensive bilateral retinal photocoagulation was deemed necessary. The procedure was performed successfully, applying 885 spots in the right eye and 1,776 spots in the left eye (Figure 3). The laser procedure was performed using a 532-nm green laser, with power settings ranging from 260 to 360 mW and a fixed exposure time of 0.3 seconds per spot. Subsequent evaluation revealed that retinal vasodilation had improved, and no progression or recurrence of retinopathy was observed. However, organic changes were identified in the macula of the left eye, including temporal dragging of the retina and macular scarring. Two years after treatment, no recurrence or relapse of retinopathy has been observed, and the right eye exhibits a satisfactory pursuit response, while the left eye demonstrates no pursuit response but retains light perception (Figure 4).

The case did not display typical dermatological signs associated with pigmentary disorders, such as vesicles or later whorled pigmentations, and genetic tests, including whole-exome sequencing and microarray comparative genomic hybridization analysis, revealed no clinically significant copy number variations or disease-related variants. Investigations for metabolic disorders and neurological abnormalities, including brain MRI, did not show any pertinent findings. Notably, the patient did not exhibit common ROP risk factors such as oxygen toxicity or cerebral hemorrhage. Although developmental delay was noted during follow-up, brain imaging did not reveal any correlating structural abnormalities.

## Discussion

Two key insights were gleaned from this case. First, this case demonstrates that severe retinal vascular dysplasia resembling AROP can develop even in moderately preterm infants, such as the present case, who are not extremely premature and, despite some baseline risk due to low birth weight, do not exhibit typical exacerbating ROP risk factors such as oxygen toxicity or cerebral hemorrhage. Second, in such cases, FA, in addition to the usual fundus examination, can be used to identify and delineate the avascular area more accurately.

It is widely acknowledged that the likelihood of developing ROP is significantly elevated in very preterm infants and infants with very low birth weight [1,2]. The reported risks of developing ROP include the use of high concentrations of oxygen, cerebral hemorrhage, blood transfusion, infection, and gastrointestinal perforation [14]. However, none of these complications were observed in the present case, despite a birth weight of 861 g. The patient did not require special systemic management after birth and differed from typical cases of ROP. This observation led to the hypothesis that the condition may be consistent with a distinct form of retinal vascular dysplasia, potentially related to an angiogenesis disorder rather than ROP.

Furthermore, severe AROP-like retinopathy has been observed to be complicated by cerebral infarction and central nervous system disorders. A correlation between cerebral and retinal microvascular abnormalities has been documented [15-17]. In the present case, although a developmental delay was observed, no abnormal findings were identified on head MRI. Consequently, we concluded that the patient’s condition was not consistent with ROP, but rather with a condition suggestive of other vascular dysplasia. Retinal photocoagulation was therefore selected as the treatment approach, instead of anti-VEGF vitreous injection.

In addition to ROP, other differential diagnoses for severe retinal vascular dysplasia include familial exudative vitreoretinopathy (FEVR), pigmentary ataxia, Norrie’s disease, and Coats’ disease [18-20]. However, in the present case, there were no skin findings characteristic of ataxia pigmentosa (blistering and later spiral hyperpigmentation), and the diagnosis of the aforementioned diseases was unlikely due to the patient’s gender and the fatal nature of ataxia pigmentosa. Furthermore, the genes responsible for abnormal retinal angiogenesis, including *FAD4*, *LRP5*, *ZNF408*, *TSPAN12*, and *KIF11*, have been associated with FEVR [21,22]. Similarly, *NDP* has been linked to FEVR and Norrie’s disease, *RCBTB1* to FEVR and Coats’ disease, and *IKBKG* to ataxia pigmentosa [21,23-25]. However, all of these were negative in the present whole-exome analysis. These findings suggest that even in infants who are not extremely premature, there is a potential for developing severe ROP-like retinal vascular dysplasia in the context of systemic anomalies.

Fundus photography is a valuable diagnostic tool for assessing retinopathy. However, previous studies suggest that FA may offer advantages over standard fundus photography, particularly in delineating the macular center and identifying lesions in ROP [26,27]. In this case, FA effectively visualized ischemic areas in both eyes, especially the left eye, enabling appropriate laser planning. Optical coherence tomography angiography, although capable of providing high-resolution vascular imaging, has practical limitations in infants due to its limited field of view and the requirement for multiple image acquisitions [28,29]. Additionally, pediatric patients often have difficulty maintaining a steady gaze, further reducing its applicability. Consequently, FA remains a more practical modality in this population. As illustrated by this case, combining FA with fundus examination allows for more accurate identification of avascular retinal zones and informs timely intervention. Continued research is essential to further clarify the mechanisms underlying atypical retinal vascular dysplasias and to optimize diagnostic and therapeutic strategies in affected infants.

## Conclusions

We present a rare case of a moderately preterm infant without traditional ROP risk factors who developed AROP-like retinal vascular dysplasia. Timely FA-guided photocoagulation was effective in halting disease progression. This case underscores the need for comprehensive ophthalmologic screening in all preterm infants and supports FA as a valuable tool in identifying avascular zones requiring treatment. Additionally, systemic anomalies observed in this patient may point to a broader vascular dysgenesis, warranting further genetic and syndromic exploration.
